# Functional foods and their potential impact on women’s health in Chile

**DOI:** 10.3389/fnut.2026.1778228

**Published:** 2026-04-30

**Authors:** Marcela Low Mansilla, Berta Henríquez Cabezas, Paola Burgos Villegas, Luisa Quesada Romero, Claudia Torres-Farfán, Esteban Salazar-Petres

**Affiliations:** 1 Escuela de Química y Farmacia, Facultad de Ciencias, Universidad San Sebastián, Valdivia, Chile; 2 Departamento de Ciencias Biológicas y Químicas, Facultad de Ciencias, Universidad San Sebastián, Valdivia, Chile; 3 Escuela de Obstetricia, Facultad Ciencias Para el Cuidado de la Salud, Universidad San Sebastián, Valdivia, Chile; 4 Escuela de Nutrición y Dietética, Facultad de Ciencias de la Rehabilitación y Calidad de Vida, Universidad San Sebastián, Valdivia, Chile; 5 Laboratorio de Cronobiología del Desarrollo, Facultad de Medicina, Instituto de Anatomía, Histología y Patología, Universidad Austral de Chile, Valdivia, Chile

**Keywords:** advanced age, bioactive compounds, Chilean woman, climacteric, feeding, pregnancy

## Abstract

Feeding transcends mere nourishment by integrating social and cultural factors. The foods we consume hold significant economic and social relevance within territories and are rich in bioactive compounds that confer health benefits. In the context of an aging population and increased life expectancy, functional foods represent a healthy alternative for Chilean women. Evidence among Chilean women suggests that incorporating functional foods into the diet, alongside modifying behaviors and risk factors such as the desynchronization of biological rhythms, can aid in preventing non-communicable chronic diseases such as obesity, diabetes, and hypertension. This review examines the potential beneficial effects that locally sourced functional foods may have on the health of Chilean women during critical life stages, including pregnancy, the climacteric, and advanced adulthood. A healthy diet incorporating functional foods commonly consumed in Chile, particularly those endemic to the country, could contribute to improved women’s health across key stages of the life course, reduce obesity rates, and exert both short- and long-term effects on the prevalence of diseases affecting older women, mothers, and their offspring.

## 1 Introduction

Eating is more than just an activity to satisfy people’s nutritional and energy needs; it is also a practice that connects with the social and cultural environment. This environment influences decisions about how and at what time of day to eat and is an integral part of human daily life (1). A balanced diet is essential to ensure optimal growth and development at all stages of life, supporting not only physical but also mental and social well-being (2). In the search for nutritional alternatives, products originating from the same territory become relevant when considered functional foods, meaning they contain bioactive compounds with health-beneficial properties (3–5). The importance of functional foods has been widely recognized, as their neuroprotective, anti-tumor, anti-inflammatory, antioxidant and prebiotic properties not only providing benefits for human health but also boosting local economic activities (6).

Given the worrying global health context, behavioral risk factors contribute to millions of deaths. In 2019, these factors were responsible for 22 million deaths worldwide, with an unhealthy diet accounting for 14% of these fatalities, ranking as the second leading factor after tobacco consumption (7, 8). Chile mirrors this global trend; in the same year (2019), behavioral risk factors contributed to 35,731 deaths (32% of total deaths) in the country. Notably, and differing from the global pattern, diet stands as the most important risk factor in Chile, responsible for 40% of deaths, followed by tobacco consumption (36%) and alcohol consumption (24%) (8). Beyond diet, other behavioral risk factors such as shift work, exposure to artificial nocturnal light, and irregular eating schedules can cause chronodisruption, an alteration in the synchronization between physiological functions and the external environment (9). In this regard, Chilean women spend daily 2 h and 5 min more than men on unpaid work activities (10), suggesting substantial differences in daily activity patterns that can affect sleep, eating, and influence overall health.

In response to this pressing national health landscape, the Ministry of Health of Chile presented the National Food and Nutrition Policy in 2018, aiming to provide guidelines for the development of programs and strategies to address food and nutrition-related problems in the country. In addition, the National Health Strategy 2020–2030 further highlights the importance of increasing food and nutritional security, adopting an approach that considers Chile’s territorial and cultural diversity, to promote healthy food access, foster sustainable environments, and prevent diseases across the life course (7).

These strategies are particularly crucial given the higher prevalence of overweight and non-communicable chronic diseases (NCDs) described in adult Chilean women. Additionally, Chile is in an advanced stage of population aging, characterized by increased life expectancy (higher in women) and a low fertility rate associated with delayed pregnancy age (11). This demographic shift presents a significant challenge for public health policies, particularly impacting the epidemiology of the female population, which already shows a high prevalence of NCDs such as diabetes and hypertension (12). To contextualize these significant health challenges, Table 1 presents a comprehensive overview of the prevalence of various risk factors for NCDs across different life stages. This data, encompassing key categories such as tobacco and alcohol consumption, mental health issues, sleep disturbances, and nutritional and metabolic status, highlights the high prevalence of these risk factors and reveals significant differences throughout the Chilean woman life course. This national health scenario highlights how functional foods and dietary interventions can play a crucial role in mitigating these risks in Chilean woman. Therefore, addressing women’s health from a life course perspective is essential, recognizing existing health differences based on context and territory.

**Table 1:** Prevalence of non-communicable disease risk factors across life stages in Chilean women.

Risk factor	Life stage	Prevalence	Reference
Tobacco	(Total: 33.3%; Woman: 29.1%; Men: 37.8%)		(12)
Daily consumption	Pregnancy	4.8%	(148)
Current consumption	Youth	22.8%	(12)
Current consumption	Adulthood	~41%	(12)
Current consumption	Climacteric	~31%	(12)
Current consumption	Older Adulthood	12.8%	(12)
Alcohol	(Total: 11.7%; Woman: 3.31%; Men: 20.5%)		(12)
Risky consumption	Youth	6.8%	(12)
Risky consumption	Adulthood	~7%	(12, 149)
Risky consumption	Climacteric	~2%	(12)
Risky consumption	Older Adulthood	0%	(12)
Mental health problems	(Total: 17.1%; Woman: 17.4%; Men: 16.8%)		(149)
Perceived loneliness	Adulthood	~22%	(149)
Mood	Adulthood	16.7%	(149)
Depression	Adulthood	17.8%	(149)
Depression	Older Adulthood	68.6%	(150)
Depressive symptoms	Postpartum	~45%	(151, 152)
Depression	Pregnancy	13.9%	(152)
Anxiety	Adulthood	34.6%	(149)
Anxiety	Pregnancy	44.3%	(152)
Anxiety	Postpartum	~47%	(152)
Professional consultation	Adulthood	43.9%	(149)
Depressive symptoms	Youth	~52%	(153)
Depressive symptoms	Adulthood	~54%	(153)
Depressive symptoms	Older Adulthood	~56%	(153)
Sleep	(Total: 26.3%; Woman: N. R.; Men: N. R.)		(154)
Insomnia	Youth and adulthood	11%	(149)
Medication use	Youth and adulthood	18.2%	(149)
Medication use	Older Adulthood	24.5%	(68)
Difficulty falling asleep	Older Adulthood	45.3%	(68)
Nutritional and metabolic status	Total obesity: 30–34%; Total overweight/ obesity: ~78% (Woman and men) Total diabetes:12.0%; Woman: 14.0%; Men: 10.6%		(155, 156)
Sedentarism	Adulthood	38.4%	(149)
Low physical activity	Adulthood	7.4%	(157)
Low physical activity	Climacteric	11.7%	(157)
Low physical activity	Older Adulthood	10.5%	(157)
Unhealthy diet	Adulthood	84%	(157)
Unhealthy diet	Climacteric	86.6%	(157)
Unhealthy diet	Older Adulthood	75.7%	(157)
Diabetes	Pregnancy	5.6%	(31)
Diabetes	Adulthood	~12%	(158)
Morbid obesity	Adulthood	4.7%	(12)
Obesity	Pre-gestational	30.1%	(159)
Obesity	Pregnancy	32.3%	(45)
Obesity	Youth	17.1%	(12)
Obesity	Adulthood	34%	(12)
Obesity	Climacteric	38.1%	(12)
Obesity	Older Adulthood	34.5%	(12)

Diet, as a principal modifiable behavioral factor, can positively impact disease prevention, making what to eat and when to eat relevant aspects when discussing women’s health. Adopting healthy eating habits improves health and helps address overweight and obesity issues, favorably affecting the well-being of women and the population (12). Against this background, this review aims to describe the potential impact on the prevention of NCDs of functional foods with territorial relevance in Chile, on women’s health across critical life stages, such as gestation, the climacteric, and older adulthood. These specific stages are chosen not solely due to the prevalence of NCDs, but primarily because they represent critical windows marked by significant physiological and hormonal changes, rendering women particularly vulnerable to risk factors including diet-related health complications and making nutritional interventions highly impactful.

## 2 Methodology

The literature search was conducted in the Pubmed and SciELO Chile databases. The filters used were “articles” in the Pubmed database and “article” in SciELO Chile with published date between 2014 and 2026. The terms used for the search were: “chilean” AND “woman” AND “nutrition” in Pubmed database, and: “mujer” AND “chilena” AND “nutrición” in SciELO Chile. In this research articles that included women girls, adolescents, and adult women, as well as pregnant women in the adult age range (18 to 45 years), women in the climacteric phase (45 to 55 years) and older adulthood (60 years and over) were considered. No results were found in these databases when the terms “c*ompuestos bioactivos chilenos*” or “chilean bioactive compounds” were added to this search. Therefore, a second search was performed using the terms “chilean bioactive compounds” in Pubmed, and “alimentos” AND “mujer” AND “Chile” in SciELO Chile. For this second search, the inclusion criteria were bioactive compounds from functional foods in Chile. Articles that considered the general population, reporting the proportion of women and men, but without detailing findings in women in the study groups, were excluded. In the case of bioactive compounds, articles without biomedical relevance were excluded.

Article references were reviewed as a second method of inclusion. All primary and secondary articles whose abstract indicated the search terminology were included. All articles written in English and Spanish or translated into these languages were analyzed. Articles considered potentially relevant were subsequently evaluated through full-text reading. As this work represents a narrative review, no formal quality assessment or risk-of-bias analysis was conducted.

## 3 Women’s health and nutrition in Chile

Globally, it has been estimated that 39% of adults are overweight and 13% are obese (13). In Chile, 39.8% of the population is estimated to be overweight, 31.2% obese, and 3.2% morbidly obese (14). Among Chilean women, there is a prevalence of 37% overweight, which has increased by 5% since 2003, and an obesity rate of 33.9%, almost 5% higher than in men. The largest increase since 2003 is in women aged 25–44, an age range where severe or morbid obesity also occurs at 4.7% (12). This significant increase in a young, fertile, and economically active group is concerning because it has short- and long-term consequences for women’s health and future generations. Therefore, aspects such as eating time, diet type, and nutritional information are relevant when discussing women’s health, as modifying these factors correctly can positively impact disease prevention.

Chilean women health has been addressed both nationally and internationally, with studies including dietary habits and the impact of nutritional learning (15–17). It has been described that Chilean women with lower nutritional knowledge have a higher prevalence of excess weight (18). Also, Chilean women with polycystic ovary syndrome present high body mass index and metabolic alterations such as dysglycemia, dyslipidemia, and a high prevalence of metabolic syndrome, suggesting an association (19). Regarding dietary habits, differences have been described according to the hormonal cycle: normal-weight Chilean women increase metabolic energy expenditure during the luteal phase and concurrently increase lipid utilization, which helps maintain energy balance, whereas obese women do not show an increase in basal metabolic expenditure in this phase but do show increased carbohydrate intake, leading to an energy imbalance that may hinder effective weight control (20).

Consequently, excess malnutrition before pregnancy is a public health challenge as it affects the health of pregnant women and newborns (16). Public policies should focus on healthy eating, nutritional monitoring in women of reproductive age (15–49 years), antenatal nutritional evaluations, and prevention and control strategies for micronutrient deficiencies such as vitamin D, for which sedentary adult women have been reported to have severe deficiencies (21, 22). These measures can impact women across pregnancy, lactation, and the climacteric stage.

NCDs, including obesity, accounted for approximately 5 million deaths worldwide in 2019. In response, the World Health Organization recommends accelerating strategies for the prevention and management of obesity across the course of life, alongside promoting healthy eating and lifestyle behaviors to reduce the burden of these diseases (23). Diet changes may also help prevent and treat endometriosis, an inflammatory condition affecting women (24). In Chile, it has been described that a 5% reduction in body weight would decrease cardiovascular risk levels in obese Chilean women by 8 to 23% (25). Chile’s 2020–2030 health strategy aims to protect against major NCDs such as diabetes, cardiovascular disease, stroke, and cancer, which were the main causes of death in Chile during 2021. Specifically, “circulatory system diseases” and “tumors (neoplasms)” accounted for 44.4% of total deaths in the population and 46.3% of deaths in Chilean women (26, 27). Therefore, promoting access to and knowledge of healthy foods is fundamental to fostering all dimensions of people’s health and well-being and to maintaining quality of life.

## 4 A life-course perspective on women’s health in Chile

From a biological perspective, women experience significant changes at various stages of their lives. However, addressing health must go beyond the biological aspect; this is where the life course perspective in women’s health becomes relevant. Considering that a woman’s vital trajectory develops biologically and socially, it responds to a historical period and relates to the cultural, territorial, economic, political, technological, and ecological framework (28). For a more comprehensive understanding of a woman’s life course, the Ministry of Health in Chile, through its life course department, emphasizes the importance of people’s relationships with their environment to promote healthy development and longevity (27). Chile is currently undergoing a demographic transition in its population profile. In general terms, there is a higher life expectancy, which is greater in women; for 2023, it was 84.1 years compared to 78.7 years in men (11). This transition is accompanied by a decrease in birth rates and a delay in motherhood (29). Over the past 40 years, there has been a reported increase from 1.6 to 6.1% of women having their first child between the ages of 36 and 40, and from 0.4 to 1.2% of women having their first child after the age of 40 (30). Moreover, gestational age is closely related to pregnancy-specific pathologies such as gestational diabetes (GD) and hypertensive syndrome, which are risk factors for developing NCDs such as diabetes and hypertension in adulthood (31). The epidemiological changes exposed, particularly the increase in life expectancy of Chilean women, emphasize the need for a public health perspective. Key life stages like gestation and the climacteric are fundamental to considering in this approach, where they impact women’s health throughout their lives.

### 4.1 Pregnancy

Gestation is a profound biological event characterized by significant metabolic and endocrine changes in women. During this period, maternal organs and systems undergo modifications primarily to ensure adequate nutrient and oxygen transfer to the developing fetus (32). These physiological adaptations, particularly in glucose and insulin homeostasis, involve organs such as the pancreas, liver, adipose tissue, and intestine (33, 34), largely driven by placental hormones to meet both maternal and fetal metabolic demands (35, 36). However, under adverse metabolic conditions like obesity or diabetes, these adaptive processes can fail, negatively affecting fetal development, increasing the risk of adult-onset diseases, and impacting the postpartum health of the mother (37, 38). Pre-gestational overweight, for instance, is a recognized risk factor for metabolic complications like insulin resistance during pregnancy and postpartum (17). Moreover, gestational metabolic diseases are linked to increased oxidative stress, release of pro-inflammatory cytokines, and altered immune processes in the placenta (39), with mitochondrial dysfunction observed at the cellular level under such adverse conditions (40, 41).

In this context, bioactive compounds from functional foods can exert diverse beneficial effects during gestation, extending beyond antioxidant activity. They can mitigate oxidative stress and may also act as immunomodulators and anti-inflammatory agents, contributing to overall maternal-fetal well-being (42–44). Therefore, proper management of woman’s metabolism and nutrition throughout gestation is necessary, as a balanced approach can prevent risks and promote the health of both mother and fetus.

In Chile, despite the existence of recognized maternal–infant health and nutrition programs, efforts have been insufficient to curb the rising rates of maternal and infant obesity. Currently, one in three pregnant women in the country is classified as obese, with regional data showing that 67% of pregnancies in the Araucanía region are affected by maternal obesity (45). Excessive weight gain during pregnancy is linked to factors such as education and a history of chronic diseases in the mother (46). Furthermore, a recent study highlights a drastic increase in the prevalence of diabetes among Chilean women, with projections that improved detection could significantly reduce births with associated pathologies by 70.4% and care costs by 67.3% (47).

In this context, maternal nutritional status and metabolic status profoundly influences infant birth weight. Varying percentages of newborns with low or high birth weight have been consistently observed, with maternal factors like Body Mass Index (BMI), gestational weight gain, preeclampsia, and GD being key predictors (48). Evidence suggests a transgenerational risk: a cohort study of 396 Chilean mothers showed that their daughters’ birth weight was associated with the mothers’ anthropometric and metabolic data 10 years postpartum (49). Similarly, in another cohort of 596 Chilean mothers, pre-gestational weight was linked to a higher risk of early adiposity rebound in children (before 5 years of age) (50).

The overall quality of the diet in the Chilean urban population (aged 15 to 65 years) is far from optimal, characterized by high consumption of trans fats, sodium, and sugary drinks (51). Women are identified as a high-risk group in this context, underscoring the urgent need for public health policy interventions to improve dietary patterns and reduce the burden of diet-related NCDs (52). Specific nutritional interventions during pregnancy include abandoning sugary drink consumption, restricting bread to no more than two portions daily, replacing red meat with white meat, and incorporating a wide variety of vegetables and fruits. Promoting breastfeeding and facilitating attendance at physical activity classes during gestation are also crucial (53). However, a study of 601 pregnant Chilean women indicated that although non-caloric sweeteners (sucralose, acesulfame, Stevia, aspartame) are highly consumed (up to 95.6%), their mild effects on maternal and infant health were observed, even within acceptable daily intake levels (54).

Another study reported an association between BMI and iron deficiency in Chilean postpartum women, showing that obese women may experience reduced iron absorption, a finding attributed by the authors to obesity-related subclinical inflammation (55). Furthermore, the quality of the mother’s diet, particularly concerning fatty acid types consumed during gestation, is associated with the fatty acid composition in red blood cell membranes and breast milk (56). In general, Chilean women consumed relatively high amounts of saturated fat, adequate amounts of mono- and polyunsaturated fatty acid (PUFA), but low intakes of n-3 long-chain PUFA. Given the critical roles of arachidonic acid (AA) and docosahexaenoic acid (DHA), both n-3 long-chain PUFA, for neonatal brain and visual development, the low maternal status is of particular concern (56).

In the search for strategies to improve breast milk quality, chia oil, rich in alpha-linolenic acid (ALA), stands out, as Chilean women consuming it have shown increased ALA intake and concentration in milk (57). However, recent findings from the Chilean women’s cohort for maternal–infant studies (CHIMINCs-II) reported an association between non-nutritive sweetener consumption (especially sucralose) and the risk of GD in pregnant women (53, 58). Therefore, comprehensive nutritional care and medical attention during gestation are not only fundamental for the immediate well-being of the mother but are also long-term investments for women’s health.

### 4.2 The climacteric phase and aging

The climacteric is a transitional stage in a woman’s life that marks the end of her reproductive period, generally occurring between 45 and 55 years of age. It is a process characterized by significant hormonal changes, mainly a decrease in estrogen and progesterone production (59). Declining estrogen levels during reproductive aging has been directly associated with the occurrence of oxidative stress, leading to increased free radical production and reduced antioxidant defenses during this period (60). This condition is further exacerbated in obese postmenopausal women (61). In addition, during this stage, women experience various physical and emotional changes, including menstrual irregularities, hot flashes and night sweats, alterations in mood, insomnia, weight gain and redistribution of body fat, decreased sexual desire, vaginal dryness, and changes in skin and hair (59). It is crucial to offer support and education to woman during the climacteric phase to address these changes and prevent future complications.

In the Chilean context, a longitudinal study (GOCS) in premenopausal women (average age 37 years) revealed a significant association between metabolic syndrome and mammographic density markers used in breast cancer detection (62). Chilean women during the climacteric phase exhibit some high risk associations with hypertension, as well as with diabetes (63). Chile’s epidemiological profile projects women’s life expectancy to reach 87.8 years by 2050, significantly higher than men’s. However, older adulthood often involves negatively self-rated health, functional deterioration, depressive symptoms, and cognitive impairment (64). Beyond the climacteric phase, an aging population necessitates attention to the diet and lifestyles. For instance, a study in southern Chile, primarily involving women (81.6%), found that only 14.1% of participants had good diet quality, with 83.8% requiring urgent improvements (65). These findings indicate an association between food insecurity, cardiovascular risk, and diet quality in Chilean woman, highlighting the relevance to modify nutrition in these later stages of life.

In Chile, approximately 1.3 million women aged 45–64 are in the climacteric stage (12). The Ministry of Health established the PACAM program (P*rograma de Alimentación Complementaria del Adulto Mayor*) in 1998, to promote healthy aging through adequate nutrition (66). However, a study of PACAM participants revealed that only 63% consumed these foods, typically fortified milk products, instant soups and cereals, with average daily intakes below 50% of the recommended portion. Significant serum deficiencies of vitamin D, B12, and calcium were observed, particularly among women consuming PACAM foods (67). This suggests that while PACAM foods contribute to micronutrient intake, they may not be sufficient to ensure adequate levels in the older adult population.

Ultimately, addressing the complex interplay of physiological changes, nutritional deficiencies, and health risks throughout the climacteric phase into older adulthood requires comprehensive nutritional and lifestyle interventions to support the metabolic health and overall well-being of Chilean women.

## 5 Chronodisruption and its impact on women’s health across the life course in Chile

Chronodisruption can significantly affect women’s health in Chile. For example, despite only 4.2% of employed Chilean women working shifts, the percentage of women reporting sleep problems reaches 26.46% compared to 17.15% in men (68, 69). Job insecurity, working from home, and being a woman are identified as risk factors associated with sleep problems (70). A study of healthcare workers in Chile, predominantly women (>90%), revealed that shiftwork impacts diet quality, eating patterns, and meal timing, though not physical activity or daytime sleepiness (71). This highlights the importance of maintain food quality and timing for individuals on rotating shifts. Epidemiological studies suggest that shift work is associated with an increased risk of chronic diseases, including diabetes and obesity (72, 73).

This circadian impairment is strongly associated with problems related to fertility and pregnancy, particularly spontaneous abortion (74, 75). During pregnancy, alterations in circadian rhythms can affect glucose homeostasis by modifying melatonin rhythms—a key hormone for sleep control, energy metabolism, and insulin secretion (76, 77). Animal models also show that exposure to altered eating schedules or artificial light at night can alter melatonin, glucose, leptin, and corticosterone production, resulting in impaired maternal physiological adaptation and altered glucose regulation (78). Thus, chronodisruption can increase the likelihood of pregnancy complications and affect the long-term health of Chilean women and their offspring.

Chronodisruption also significantly impacts women during the climacteric and later stages of life. A bidirectional relationship exists between altered sleep and vasomotor symptoms, creating a cycle of sleep deprivation and hormonal dysregulation that negatively affects the health and quality of life of menopausal women (79). Circadian regulation of body temperature is closely linked to menopausal symptoms such as hot flashes and night sweats, as estrogen fluctuations dysregulate the thermoregulatory system (80). The decrease in estrogen and progesterone hormone levels further disrupts biological rhythms, potentially aggravating health and quality of life in older adulthood (81, 82).

In Chile, older women between 60 and 64 years of age show an increase in shift work (day and night), often coinciding with a peak in domestic tasks, possibly linked to the care of older individuals (83). Furthermore, a study conducted in two Chilean cities, Temuco and Valdivia, found that poor sleep quality predicts greater pain in individuals with musculoskeletal disorders, with higher prevalence observed among women (84). Reduced sleep efficiency and alteration of circadian rhythms contribute to the development of chronic diseases and functional decline, representing critical challenges in the aging Chilean population.

Incorporating functional foods with chronoregulatory properties into the diet emerges as a potential strategy against metabolic alterations associated with circadian disruption (85). In the Chilean context, several territorially relevant foods may contribute to this effect. Quinoa (*Chenopodium quinoa*) possesses the biosynthetic machinery to produce melatonin, suggesting its potential as a dietary source of this chronobiotic (86). Piñones and salmon are also recognized as foods with relevant melatonin and tryptophan content, the latter being a biochemical precursor of serotonin and melatonin whose dietary availability has been associated with improved sleep quality and circadian regulation (87). Additionally, native berries such as maqui (*Aristotelia chilensis*) and murta (*Ugni molinae*), rich in polyphenols including anthocyanins and flavonols, may indirectly modulate the circadian system by influencing melatonin levels and clock gene expression (85, 88). Altogether, these foods act as modulators of the nutrition–circadian axis, where dietary melatonin, tryptophan, and antioxidant compounds may contribute to improved sleep quality and temporal regulation of physiological processes.

## 6 Functional foods with territorial relevance in Chile: biomedical properties and implications for chronic disease prevention

Functional foods are those that contain significant amounts of bioactive compounds, such as polyphenols, phytosterols, omega-3 fatty acids, and dietary fiber, which provide health benefits beyond their basic nutritional value (3). These compounds act at the cellular level, modulating inflammatory processes, reducing oxidative stress, improving intercellular communication, regulating gene expression, and strengthening the immune system. Regular consumption of functional foods contributes to the prevention of NCDs such as type 2 diabetes, hypertension, and cardiovascular diseases (89, 90).

In the Chilean context, several locally relevant functional foods such as berries (blueberries, maqui, and murta), quinoa, honey, cochayuyo, chilote garlic, pine nuts, chestnuts and chañar - are rich in these bioactive compounds. The composition and concentration of bioactive compounds in these foods are strongly influenced by the conditions of the territory in which they grow, including soil characteristics, water availability, altitude, and other environmental factors.

This intrinsic link between geo-climatic conditions and the biochemical profile of indigenous foods is particularly pronounced in Chile’s diverse ecosystems, directly shaping both quantity and quality of beneficial compounds in local flora and fauna, and enhancing their unique therapeutic potential. For example, the antioxidant capacity of berries varies depending on the region in which they were cultivated, as shown for crops from the Araucanía and Los Ríos regions (91). Similarly, native Chilean honey exhibits antimicrobial, antioxidant, and anti-inflammatory properties that are strongly influenced by the biodiversity of the local flora and environmental conditions of the territory where it is produced (92).

Chile, due to its geography and climate, is a country rich in endemic species that exist only in specific geographical locations and can be part of a daily diet. However, many times the foods found in the diet come from other parts of the world, but over time they can also generate a close cultural and economic relationship with territory. The territorial relevance of local species is due to their biomedical properties and their ancestral use by indigenous people (93). These properties depend largely on the presence of bioactive compounds that can modulate cellular function in tissues, organs, and systems.

The 2023 National Food Sovereignty Strategy report from the Ministry of Agriculture (94) mentions that diets rich in functional foods are particularly important for vulnerable socioeconomic groups, women, girls, children, and older adults. The strategy proposes that healthy diets should be promoted in sustainable food environments, and its action lines propose improving data and statistics on food consumption by socioeconomic level, gender, age range, territory, etc. Since the beginning of the COVID-19 pandemic, there has been a significant change in people’s perception of health and nutrition, positioning them as central axes in disease prevention and strengthening the immune system (95). This social change has driven increased demand for value-added products, of local origin, and produced under sustainability criteria, reflecting a transition toward more conscious consumption patterns. In this context, incorporating functional foods into the diet can generate long-term positive effects by acting at the molecular level on the physiology of NCDs. In Chile, functional foods with significant economic value have been reported in different regions, associated with their local production, commercialization, and consumption (96). Nevertheless, several functional foods are still in early research stages, with their potential biomedical properties primarily documented through local knowledge and community reports. Activities aimed at promoting and educating about the consumption of functional foods in various regions can not only increase their commercial value but also strengthen related economic activities and contribute to improving population health.

## 7 Functional foods with territorial relevance in Chile: bioactive compounds and health implications for women

Diet plays a central role in human health, influencing physiological and metabolic outcomes beyond basic nourishment. Aligning food intake with circadian rhythms is important for maintaining metabolic homeostasis, particularly during key stages of a woman’s life such as pregnancy, the climacteric, and older adulthood (88, 97–99). Functional foods contain bioactive compounds which are low concentration molecules that interact with molecular targets to modulate inflammation, oxidative stress, gene expression, intercellular communication, and immune responses (100).

These compounds include phytochemicals such as flavonoids (quercetin, catechins), anthocyanins (delphinidin, cyanidin), tannins, betalains, carotenoids (*β*-carotene, lutein), plant sterols, and glucosinolates, many of which exhibit antioxidant and anti-inflammatory properties (101). Dietary fiber supports the intestinal microbiota, while prebiotics (inulin, fructooligosaccharides) and probiotics (Lactobacillus, Bifidobacterium) contribute to immunometabolic health. Vitamins (C, E) and minerals (selenium, zinc) also act as cofactors in redox reactions and antioxidant defenses (102). Animal-derived foods provide additional bioactives, including omega-3 fatty acids (EPA and DHA), conjugated linoleic acid (CLA), carotenoids such as lutein, and bioactive peptides derived from milk proteins with antihypertensive, immunomodulatory, and antithrombotic effects (103).

Regular consumption of these functional foods, particularly when aligned with circadian rhythms, has been associated with the prevention of metabolic, cardiovascular, and bone disorders, as well as non-communicable diseases such as type 2 diabetes and hypertension (89, 90).

### 7.1 Functional foods of territorial relevance in Chile

In the Chilean context, these principles are particularly relevant, as the country harbors a wide variety of locally available foods rich in bioactive compounds. These include berries, native honey, nuts, quinoa, chilote garlic, manzana limona, cochayuyo, chañar, among others. While some are supported by strong scientific evidence, others continue to be used mainly based on the long-standing traditional knowledge of local communities.

The composition of these functional foods is strongly associated with the territory in which they develop, including factors such as soil characteristics, water availability, and altitude. This intrinsic relationship between geo-climatic conditions and the biochemical profile of local foods is particularly evident in Chile’s diverse ecosystems, contributing to their unique nutritional and functional properties. Similarly, native Chilean honey exhibits antimicrobial, antioxidant, and anti-inflammatory properties that vary depending on the biodiversity of the flora and environmental conditions of the territory where it is produced (92).

The unique bioactive composition of these territorial foods, shaped by Chile’s diverse ecosystems, makes them particularly valuable for supporting women’s health across key stages of the life course. Increased intake of foods rich in bioactive compounds may support maternal health during pregnancy, help mitigate metabolic and inflammatory changes associated with menopause, and promote healthy aging in older women. Encouraging regular consumption of these foods may be a valuable strategy to prevent chronic diseases and promote long-term metabolic and overall health in women.

#### 7.1.1 Berries (blueberry, Maqui, and Murta)

Chilean berries such as murta (*Ugni molinae*), maqui (*Aristotelia chilensis*), and blueberry (*Vaccinium corymbosum*) are rich sources of diverse polyphenols, particularly anthocyanins. Beyond anthocyanins, these berries also contain significant levels of flavonols (e.g., quercetin and myricetin glycosides), phenolic acids (including chlorogenic, caffeic, and gallic acids), ellagitannins, and proanthocyanidins (104). This complex phytochemical matrix contributes to their high total phenolic content and underlies their strong antioxidant capacity and characteristic red-purple pigmentation, as well as their documented bioactivities related to cardiometabolic and inflammatory processes associated with the prevention of non-communicable diseases (NCDs) (105, 106).

Anthocyanins act as efficient electron donors, directly scavenging reactive oxygen species (ROS) and thereby reducing oxidative damage to lipids, proteins, and DNA, a key driver of chronic disease pathogenesis. In addition, these compounds modulate redox-sensitive signaling pathways, including activation of nuclear factor erythroid 2–related factor 2 (Nrf2), which enhances endogenous antioxidant defenses, and inhibition of nuclear factor kappa B (NF-κB), leading to reduced transcription of pro-inflammatory cytokines and adhesion molecules involved in vascular inflammation and atherogenesis (107).

These mechanisms contribute to improved endothelial function by increasing nitric oxide bioavailability and attenuating vascular inflammation, processes central to cardiovascular health (108). Emerging evidence also suggests that anthocyanin metabolites produced by the gut microbiota can influence microbial composition and host metabolic pathways, further linking berry intake to systemic anti-inflammatory and metabolic benefits (109, 110). Consistent with this, clinical and epidemiological studies have associated increased consumption of anthocyanin-rich berries with improvements in lipid profiles, cardiometabolic risk factors, and inflammatory markers. Notably, a randomized controlled trial demonstrated that daily blueberry consumption for 12 weeks improved endothelial function in postmenopausal women with above-normal blood pressure, an effect attributed to reduced oxidative stress (109, 110). Although the bioavailability of anthocyanins remains a challenge and their health effects likely involve both parent compounds and microbiota-derived metabolites, the documented anthocyanin and polyphenol profiles of Chilean berries such as maqui and murta are comparable to those of well-studied species (111, 112), suggesting similar cardiometabolic benefits. Regular consumption of these native berries may therefore support endothelial function, regulate lipid metabolism, and reduce oxidative stress and inflammation, particularly during pregnancy and the climacteric, contributing to vascular and metabolic health across key life stages in women.

#### 7.1.2 Native Chilean honey

Native Chilean honey is an important functional food due to the close link between its bioactive profile and Chile’s unique biodiversity. The country’s extensive latitudinal range, diverse climates, and high levels of endemism provide bees access to a wide variety of native floral sources, directly influencing the honey’s chemical composition and biological activity. Monofloral and multifloral honeys from endemic species produce a distinctive phytochemical fingerprint that differentiates Chilean honey from honeys produced elsewhere (92, 113).

A key feature of Chilean honey is its high content of polyphenolic compounds, including flavonoids and phenolic acids, which largely account for its strong antioxidant capacity (114). Polyphenols inhibit lipid peroxidation by scavenging reactive oxygen species (ROS), chelating transition metals, and interrupting free radical chain reactions, protecting cellular membranes and lipoproteins from oxidative damage (115). These effects are particularly relevant for preventing chronic conditions such as cardiovascular disease, metabolic syndrome, and neurodegenerative disorders (116).

In addition to antioxidant activity, Chilean honey exhibits significant anti-inflammatory effects. Its polyphenolic fraction modulates intracellular signaling pathways, downregulating pro-inflammatory cytokines such as tumor necrosis factor-alpha (TNF-*α*), interleukin-1 beta (IL-1β), and interleukin-6 (IL-6), partly through inhibition of NF-κB and attenuation of oxidative stress–mediated signaling cascades (92, 117). Chilean honey also demonstrates potent antimicrobial activity, resulting from its low pH, high osmolarity, hydrogen peroxide production, and bioactive phenolics, supporting traditional uses in wound healing and infection control (118).

The honey’s functional properties are closely tied to its botanical origin and environmental conditions, including soil composition, climate, altitude, and native plant diversity, all of which influence nectar composition and the resulting phenolic profile. Characterizing honey by floral and geographic origin is therefore essential to understand variability in its bioactive potential.

For women’s health, regular consumption of native Chilean honey may provide benefits across key life stages. Its polyphenolic compounds enhance antioxidant defenses, reduce low-grade inflammation, and support immune function. Although honey has a high glucose and fructose content, preclinical and clinical evidence indicates that it produces a lower rise in plasma glucose levels compared to refined sugars, an effect attributed to its fructose fraction and polyphenolic compounds that may modulate hepatic glucokinase activity and reduce oxidative stress (119). However, this evidence derives predominantly from animal models and small clinical trials conducted in non-pregnant adults, and no studies to date specifically support the inclusion of honey in the diet of pregnant women for glycemic management. Honey consumption during pregnancy, particularly in women at risk of gestational diabetes, should therefore be approached with caution. Beyond glycemic considerations, its anti-inflammatory and antimicrobial properties may contribute to overall wellness during the climacteric and older adulthood. Honey may also support women’s reproductive health by enhancing fertility, protecting reproductive tissues, mitigating the effects of xenoestrogens, and helping manage gynecological disorders (120). Including native Chilean honey in the diet may thus represent a complementary strategy to support antioxidant and vascular health across a woman’s life course, provided its consumption is appropriately contextualized within each life stage.

#### 7.1.3 Seaweeds (Cochayuyo)

Seaweeds constitute an important yet underrecognized component of Chile’s marine biodiversity with increasing biomedical relevance. Among them, cochayuyo (*Durvillaea antarctica*), widely distributed along the Chilean coastline from Coquimbo to Cape Horn, has been traditionally consumed since pre-Hispanic times and remains a culturally significant food. Beyond its culinary value, cochayuyo has attracted scientific attention due to its high concentration of bioactive compounds (121).

Chemically, *D. antarctica* is rich in sulfated polysaccharides, particularly fucoidans and alginates, as well as iodine, calcium, dietary fiber, and phytosterols. Fucoidans from brown algae have been reported to exert immunomodulatory and anti-inflammatory effects through modulation of cytokine production and regulation of signaling pathways such as NF-κB. Alginates and soluble fibers contribute to cardiometabolic benefits, including improved glycemic control, reduced lipid absorption, and enhanced satiety. In addition, seaweed-derived polysaccharides can function as prebiotic substrates, promoting beneficial shifts in gut microbiota composition and increasing short-chain fatty acid production (122).

The mineral profile of cochayuyo, particularly its high iodine content, also supports thyroid function and metabolic regulation, although intake must be balanced to avoid excessive consumption. Collectively, these properties position cochayuyo as a functional marine food with potential applications in metabolic health, immune regulation, and preventive nutrition (123), as well as an anti-aging food (124). As part of Chile’s natural heritage, it represents a promising example of how traditional foods can contribute to contemporary biomedical and nutraceutical research.

For women’s health, regular consumption of cochayuyo may provide significant benefits across key life stages, although specific clinical studies in women are currently lacking. Based on its bioactive composition, during pregnancy, its high fiber and polysaccharide content could support glycemic control and lipid metabolism, helping to maintain metabolic homeostasis. In the climacteric and menopause, when hormonal and metabolic changes increase the risk of cardiovascular and bone disorders, cochayuyo’s iodine may support thyroid function and hormonal balance, while fucoidans and polyphenols exhibit antioxidant and anti-inflammatory effects that could help reduce oxidative stress and systemic inflammation. Together, these bioactive compounds have the potential to promote gut, metabolic, and cardiovascular health, suggesting that cochayuyo is a promising functional food for resilience and long-term wellness in women.

#### 7.1.4 Native nuts and seeds (Piñones, castañas, quinoa)

In southern Chile, traditional plant foods such as Piñones (pine nuts) from the native Araucaria tree (*Pinus araucana*) and Castañas (chestnuts) (*Castanea* spp.) are valued for their rich nutritional and bioactive profiles. Pine nuts are abundant in monounsaturated and polyunsaturated fatty acids, tocopherols (vitamin E), and phytochemicals such as pinolenic acid, which have been associated with improved lipid metabolism, reduced inflammation, and lower cardiovascular risk (125–127). Chestnuts, although lower in fat, provide predominantly unsaturated fatty acids, tocopherols, flavonoids, potassium, and dietary fiber, contributing to antioxidant activity, blood pressure regulation, and metabolic health (128, 129).

Quinoa (*Chenopodium quinoa* Willd.), a staple pseudocereal of the Andean region, complements these nuts by providing high-quality protein, dietary fiber, flavonoids, essential minerals such as magnesium, and its own profile of unsaturated fatty acids and tocopherols. Its bioactive compounds, including polyphenols and saponins, exhibit antioxidant and anti-inflammatory effects, supporting cellular redox balance and metabolic regulation. Consumption of quinoa has been linked to improvements in lipid profiles, reductions in serum triglycerides and total cholesterol, and enhanced glycemic control, likely through combined effects on lipid absorption, insulin sensitivity, and metabolic signaling pathways (130).

From a women’s health perspective, although there is no direct evidence specifically assessing piñones or chestnuts, nut consumption in general has been shown to reduce the burden of metabolic syndrome during menopause (131). Similarly, quinoa has demonstrated beneficial effects in experimental models; for example, it ameliorates high-fat diet–induced obesity in female mice by modulating gut microbiota and adipogenesis (132).

Incorporating these traditional foods, which are rich in unsaturated fatty acids, fiber, antioxidants, and minerals, may help counteract cardiometabolic changes associated with the climacteric, such as dyslipidemia, insulin resistance, and elevated blood pressure. Regular consumption of these plant foods could therefore serve as a practical nutritional strategy to support cardiovascular and metabolic health in women.

#### 7.1.5 Native garlic (Chilote garlic)

Chilote garlic (*Allium sativum L.* var. *“Chilote”*) is a traditional variety native to the Chiloé Archipelago in southern Chile, recognized for its strong aroma, large cloves, and high content of bioactive compounds. It is particularly rich in organosulfur compounds, including allicin, as well as flavonoids and phenolic acids, which confer antioxidant, antimicrobial, and cardiovascular health–promoting properties (133).

Allicin and related sulfur compounds have well-documented vasodilatory, antihypertensive, and antioxidant effects that support cardiovascular protection. Mechanistically, allicin enhances endothelial nitric oxide (NO) production, promoting vasodilation, reducing arterial stiffness, and improving blood flow. Its antioxidant activity also scavenges reactive oxygen species (ROS) and boosts endogenous enzymes, such as superoxide dismutase and glutathione peroxidase, preventing lipid peroxidation and atherogenesis (134).

Beyond vascular benefits, organosulfur compounds in Chilote garlic exert anti-inflammatory and antithrombotic effects, further supporting cardiovascular health. Regular consumption has been associated with modest reductions in blood pressure, improvements in lipid profiles, and protection against endothelial dysfunction in experimental and clinical studies, highlighting its potential as a functional food (135).

Chilote garlic may be particularly beneficial for women during life stages marked by increased cardiometabolic risk, such as the climacteric. The decline in estrogen during this period contributes to endothelial dysfunction, elevated blood pressure, adverse lipid changes, and increased oxidative stress. The vasodilatory, antioxidant, anti-inflammatory, and antithrombotic effects of Chilote garlic can help mitigate these changes, supporting vascular function and reducing cardiovascular risk. Incorporating this traditionally cultivated garlic into the diet represents a practical and culturally relevant strategy for promoting cardiovascular well-being in women.

#### 7.1.6 Native fruits (Manzana Limona)

The apple (*Malus domestica*) is a widely consumed fruit valued not only for its taste and nutritional content but also for its rich profile of bioactive compounds. Apples are particularly abundant in polyphenols, including flavonoids (quercetin, catechins, epicatechin), dihydrochalcones (phlorizin), phenolic acids (chlorogenic and caffeic acids), and procyanidins, concentrated mainly in the peel but also present in the flesh. They are also a good source of dietary fiber, which supports gut health and glycemic control. Together, these compounds exhibit antioxidant, anti-inflammatory, and cardiometabolic regulatory properties, contributing to the prevention and management of chronic diseases (136–138).

The manzana limona is an endemic apple variety from the Los Ríos region in southern Chile, valued both for its culinary uses and cultural significance. Beyond being a traditional ingredient in regional cuisine, it represents a functional food of territorial relevance, reflecting the unique soil, climate, and biodiversity of its area of cultivation. Although its complete phytochemical profile has not yet been fully characterized, its traditional use highlights its potential as a source of bioactive compounds.

Phlorizin, a characteristic flavonoid found in apples, exhibits multiple bioactive effects, including antioxidant, anti-inflammatory, and antidiabetic properties. It modulates intestinal glucose absorption, improves lipid metabolism, and reduces oxidative stress, contributing to overall metabolic and cardiovascular health (139). Evidence also suggests that phlorizin may protect against postmenopausal bone loss by supporting bone mineral density and reducing osteoporosis risk, while consumption of phlorizin-rich fruits during lactation may help mitigate systemic inflammation (140, 141).

Similarly, quercetin, another flavonoid abundant in apples, supports women’s reproductive health. It may benefit gynecological conditions such as polycystic ovary syndrome, premature ovarian failure, endometriosis, recurrent miscarriage, and ovarian, cervical, and endometrial cancers. Quercetin modulates oxidative stress, inflammation, hormonal balance, and cell proliferation, complementing the health-promoting effects of phlorizin and underscoring apples as a source of bioactive compounds with broad relevance for women’s health (142, 143).

The combined antioxidant effects of apple polyphenols and flavonoids may also help mitigate oxidative stress associated with hormonal changes, which contributes to increased cardiometabolic risk during the climacteric (138, 144). In this context, promoting traditional varieties such as manzana limona is valuable not only for cultural and agricultural reasons but also as a strategy to encourage the consumption of functional foods with potentially beneficial bioactive properties.

#### 7.1.7 Chañar

Chañar (*Geoffroea decorticans*), a native fruit from northern Chile, is traditionally valued for its nutritional and medicinal properties. Rich in mucilages and phenolic compounds, it provides soothing effects on mucous membranes while exerting antioxidant and anti-inflammatory activity, supporting respiratory health and helping to mitigate oxidative stress and inflammation (145–147).

Although direct evidence on chañar in women is currently lacking, its demonstrated antioxidant, anti-inflammatory, and mucilage-mediated protective effects suggest potential benefits across key stages of a woman’s life. These bioactivities could be extrapolated to support maternal health during pregnancy, mitigate oxidative stress and inflammation associated with the climacteric, and promote overall metabolic and vascular resilience in older adulthood. Thus, chañar may represent a promising functional food for enhancing women’s health, even if further studies are needed to confirm its effects in female populations.

Due to their diverse bioactive compounds, the Chilean functional foods described may play a relevant role in supporting women’s health across different life stages. Although direct scientific evidence is not yet available for all of them, their documented antioxidant, anti-inflammatory, metabolic, and hormonal regulatory properties suggest potential benefits for conditions affecting women throughout the life course. Promoting their consumption also reinforces the territorial value of locally produced foods, highlighting their potential to deliver added nutritional, cultural, and economic benefits.

To synthesize this information, Table 2 provides a comparative overview of Chilean functional foods, summarizing their key bioactive compounds, recognized health benefits, and the life stages during which their consumption may be most relevant. This table serves as a concise reference illustrating how these territorially significant foods can support women’s well-being while valorizing local products and enhancing their functional and economic value.

**Table 2:** Comparison of Chilean functional foods, benefits, and recommended stages.

Region of Chile	Functional food	Bioactive compounds	Health properties	Recommended stage in women	Refs
Northern Zone (Arica - Atacama) Highland	Quinoa	Proteins, fiber, flavonoids, magnesium, iron	Antioxidant, glycemic and cardiovascular metabolism regulator	Pregnancy, fertile age	(130, 132)
	Carob	Polyphenols, tannins, low glycemic sugars	Antioxidant, prebiotic, digestive	All stages	(160)
	Chañar	Mucilages, Phenolic compounds	Anti-inflammatory, respiratory protective	Climacteric, fertile age	(146)
Central Northern Zone (Coquimbo - Valparaíso)	Chilean Papaya	Papain, carotenoids, vitamin C	Digestive, antioxidant, immunostimulant	Pregnancy, all stages	(161)
	Olive and olive oil	Monounsaturated fatty acids, polyphenols	Cardioprotective, anti-inflammatory	Climacteric, fertile age	(162)
Central Zone (RM - Maule)	Grapes	Resveratrol, anthocyanins, tannins	Antioxidant, cardioprotective, anti-inflammatory	Climacteric	(163)
	Multifloral Honey	Phenolic compounds, enzymes, flavonoids	Antimicrobial, immunomodulatory, cicatrizant	Pregnancy, fertile age	(114, 120)
Southern Zone and Austral Zone (Ñuble - Magallanes)	Murta, maqui, calafate	Anthocyanins, flavonoids, vitamin C	Potent antioxidant, neuroprotective, anti-inflammatory	Pregnancy, Climacteric	(106, 107, 164)
	Pine Nuts (Araucaria)	Unsaturated fatty acids, fiber, minerals	Energetic, cholesterol and glucose regulator	Climacteric, fertile age	(127)
	Chilote Garlic	Organosulfur compounds (allicin, ajoene)	Antibacterial, antihypertensive, cardioprotective	Climacteric	(134)
	Salmon	Omega-3 fatty acids (EPA, DHA)	Anti-inflammatory, neuroprotective, cardioprotective	Pregnancy, Climacteric	(165)
	Cochayuyo and other seaweeds	Sulfated polysaccharides, iodine, calcium, phytosterols	Antioxidant, digestive, cholesterol regulator, thyroid health	Climacteric	(121, 123)

## 8 Conclusion

In Chile, healthy eating has been promoted and supported through public health education for decades, a commitment recently reinforced by the National Food Sovereignty Strategy (2023) and the Implementation Plan of the Chilean Food-Based Dietary Guidelines (2024) issued by the Ministry of Health. However, despite these efforts, a large proportion of the population still struggles to adhere to these recommendations. Overweight and obesity remain highly prevalent among women of reproductive age, and malnutrition due to excess weight carries significant implications for key life stages such as pregnancy, the climacteric, and older adulthood, with potential long-term consequences for society.

**Figure 1:**
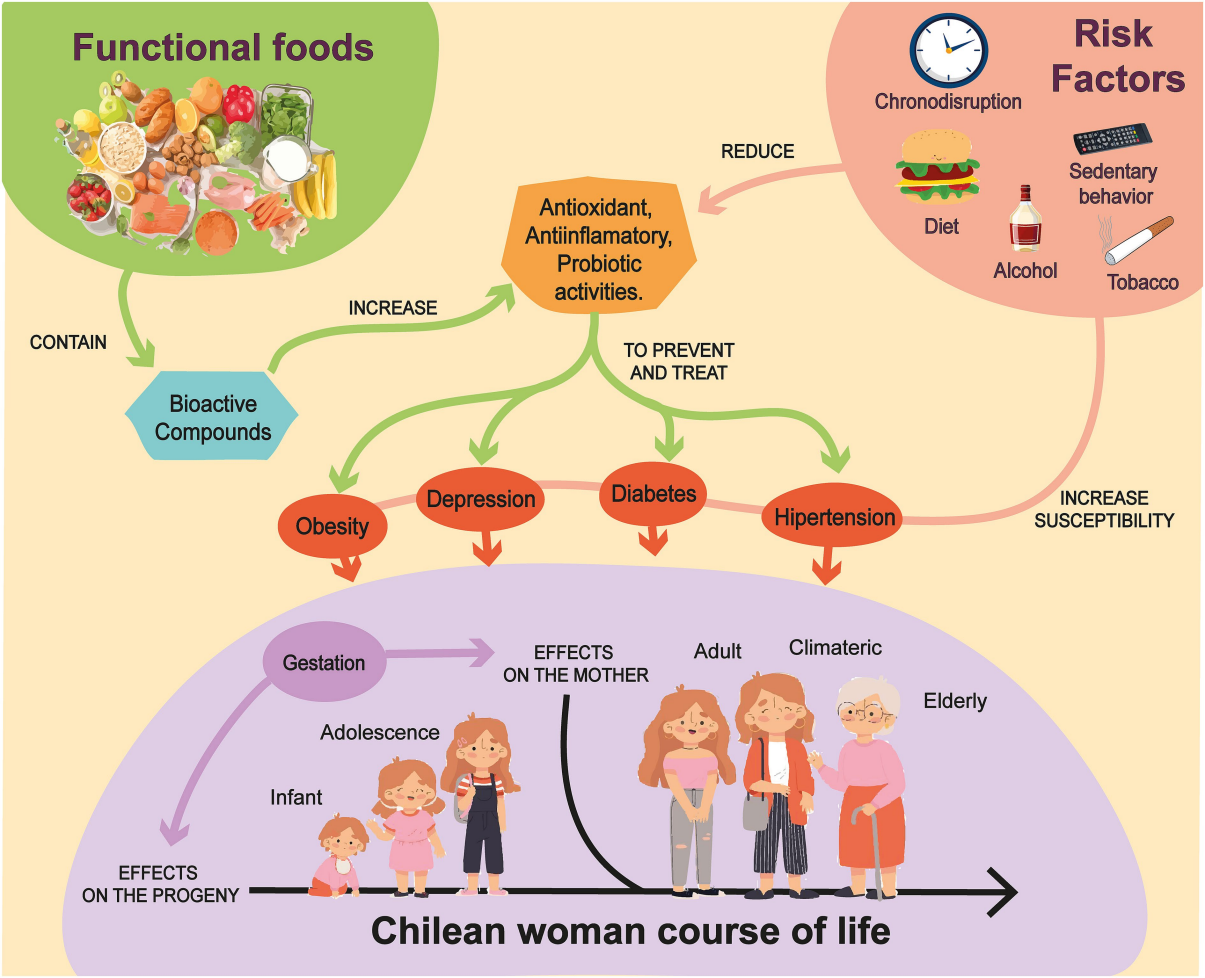
Schematic overview. Functional foods of territorial origin contain bioactive compounds with biomedical properties, including antioxidant, anti-inflammatory, and probiotic activities. The consumption of these foods may contribute to the prevention and management of diseases such as obesity, diabetes, and depression, which are prevalent across different stages of the life course of Chilean women. Susceptibility to these conditions is influenced by exposure to common risk factors in Chilean society, including chronodisruption, dietary patterns, sedentary behavior, alcohol consumption, and tobacco use. Pregnancy emerges as a critical window during which adverse exposures may have long-term effects on both maternal and offspring health.

Based on the evidence presented in this review, promoting the consumption of functional foods with territorial relevance from early stages of the female life course appears to be a promising strategy to help prevent health complications during pregnancy and the climacteric stages. Education and promotion of these foods during critical periods could act as protective factors during gestation, with potential benefits for offspring health and for healthy aging in women (Figure 1). Furthermore, advancing research on the biomedical properties of functional foods native to Chile is expected to contribute to their valorization in both local and international markets, thereby strengthening associated economic activities. Ultimately, fostering a food approach with a territorial focus—considering that women exhibit a higher prevalence of non-communicable chronic diseases—could represent an effective strategy to improve the quality of life of Chilean women.
